# Editorial: The neuron-glia crosstalk and beyond

**DOI:** 10.3389/fncel.2023.1159597

**Published:** 2023-03-02

**Authors:** Jinte Middeldorp, Rogier Min, Joseph Benetatos, Liviu-Gabriel Bodea

**Affiliations:** ^1^Department of Neurobiology and Aging, Biomedical Primate Research Centre, Rijswijk, Netherlands; ^2^Department of Child Neurology, Amsterdam Leukodystrophy Center, Emma Children's Hospital, Amsterdam University Medical Centers, Amsterdam Neuroscience, Amsterdam, Netherlands; ^3^Department of Integrative Neurophysiology, Center for Neurogenomics and Cognitive Research, Amsterdam Neuroscience, Vrije Universiteit Amsterdam, Amsterdam, Netherlands; ^4^Department of Biological Engineering, Massachusetts Institute of Technology (MIT), Cambridge, MA, United States; ^5^Clem Jones Centre for Ageing Dementia Research, Queensland Brain Institute, The University of Queensland, Brisbane, QLD, Australia

**Keywords:** glia, microglia, intercellular crosstalk, Schwann cell, astrocytes

Recent discoveries in basic and translational neuroscience led to an increased interest in understanding how different cell types in the brain work together to maintain healthy brain activity. For the brain to accomplish its many complex roles, neurons and glia must communicate with and integrate signals from the surrounding cells and environment. At the same time, disruptions in the neuron-glia crosstalk contribute to various pathological states of the nervous system, ranging from Alzheimer's disease (AD) to axon injury. Improving our knowledge of these brain intercellular interactions is therefore essential for understanding brain physiology in health and disease.

The communication between different brain cell types is established through direct (or physical) cell-to-cell interactions or by intermediates (such as molecules or vesicles acting as intercellular messages). One prime example of physical neuron-glia interactions is the close association between astrocytes and neuronal synapses at the so-called tripartite synapse (Araque et al., 1999). Modifications in this association are discussed in this Research Topic in a systematic review centered on the role of astrocytes in synapse loss associated with AD (Hulshof et al.). Furthermore, in an electron microscopy-based study of the tripartite synapse, it was revealed that the fear memory-induced synaptic activation reduces the interaction between the perisynaptic astrocyte process and the presynaptic but not the postsynaptic neuronal component in wild-type mice (Kater et al.). It would be interesting to apply a similar methodology to characterize the less-known components of the synapse: the extracellular matrix (Dityatev and Rusakov, 2011) and microglial processes touching the synapse (Schafer et al., 2013). Indeed, an integrative assessment of the synapse, essentially a complex “multi-partite” structure involving different brain cell types, may reveal novel aspects of synapse physiology.

Besides the physical neuron-glia cellular interactions, intercellular crosstalk between different types of brain cells requires the release, recognition, and uptake of molecules or other cellular components. However, this type of crosstalk can also be expanded to pathological conditions. For example, injured axons can regenerate through a complex mechanism that, among others, requires reprogramming Schwann cells to clear myelin debris, recruit immune cells, and guide the regenerating axons to their targets by secretion of neurotrophic factors and other contact-mediated signaling events. In our Research Topic, it was described a novel mechanism by which neurons release ATP that is recognized by Schwann cells, triggering the secretion of extracellular vesicles (Saquel et al.). In addition, it was found that the cargo load within these extracellular vesicles changes to support neurite growth (Saquel et al.). In another study, Dectin-1 (a molecule encoded by the *CLEC7A* gene and specific for cells of myeloid origins, such as microglia and macrophages) was found to contribute to debris clearance, nerve regeneration and angiogenesis in a nerve crush paradigm in mice (Hsu and Hsieh). These findings add to the pre-existing knowledge on neuron-glia communication and could point to novel treatment avenues for neurological conditions.

Various *in vitro* and *in vivo* models and methodologies are employed to study the neuron-glia crosstalk. As summarized in a review focusing on neuron-astrocyte involvement in synaptic removal during AD (Hulshof et al.), to investigate this crosstalk *in vitro*, researchers are using primary cell cultures isolated from wild-type or AD animal models, human embryonic stem-cell derived or human-induced pluripotent stem cell (iPSC)-derived neuron-astrocyte co-cultures treated with AD-relevant molecules or presenting with AD-associated mutations. A second review explores the advantages and disadvantages of studying microglial cell cultures ranging from ontogenically transformed stable cell lines to iPSC-derived microglial cells and 3D organoids (Aktories et al.). Within their original research article, Saquel et al. used Schwann cell primary cultures, as well as dorsal root ganglia explants and co-cultures of Schwann cells with sensory neurons of rat origin. However, it must be noted that, although some aspects of the neuron-glia crosstalk can be modeled *in vitro*, other aspects are more challenging to be studied under these conditions (such as the presence of functionally mature multi-partite synapses). An alternative model of studying neuron-glia communication can be represented by slice cultures that are positioned between the *in vitro* disadvantages and *in vivo* advantages (Aktories et al.). However, these cultures could incur high maintenance costs and necessitate a high level of expertise. In addition, perhaps still an underestimated active player involved in the crosstalk between different brain cell types, an active circulation is to date still lacking in even the most advanced culture models.

The technological advancements of the last decade for the *in vivo* study of neuron-glia crosstalk have caused substantial growth in animal models, from those expressing fluorescently marked cell types facilitating the tracing of cellular processes to the development of chimeric or humanized mouse models. The importance of this is further highlighted by the fact that most articles in this Research Topic have a major *in vivo* component. Besides the review focusing on the neuron-astrocyte communication (Hulshof et al.), two of the original research articles comprise *in vivo* analysis as the major tool of their work: the electron-microscopy study revealed the intricacies of neuron-astrocyte interactions at the synapse of wild-type or APP/PS1 mouse model of AD undergoing behavioral tasks (Kater et al.), whereas the role of Dectin-1 was investigated in wild-type mice after sciatic nerve crushing surgeries (Hsu and Hsieh).

Although sometimes more difficult to be accessed, human post-mortem tissues can also be of use to study the neuron-glia interaction. Microscopy, biochemistry, and protein expression studies can provide further insights into brain cell-to-cell communication. Hulshof et al. remark that by correlating information from neuropsychological tests with post-mortem biochemical tests, a more comprehensive overview of the role of the astrocytes on synaptic numbers can be obtained, and this can hold true for any other type of neuron-glia communication.

Taken together, the articles under the umbrella of this Research Topic showcase the complexity of the neuron-glia crosstalk that occurs throughout the central nervous system. Although we have made considerable advances in understanding how different brain cell types work together, there is still much to be revealed, holding great potential for the treatment of various neurological conditions.

## Author contributions

L-GB drafted the initial manuscript. All authors reviewed and edited the manuscript. All authors contributed to the article and approved the submitted version.
